# Ribulose-1,5-bisphosphate regeneration in the Calvin-Benson-Bassham cycle: Focus on the last three enzymatic steps that allow the formation of Rubisco substrate

**DOI:** 10.3389/fpls.2023.1130430

**Published:** 2023-02-16

**Authors:** Maria Meloni, Libero Gurrieri, Simona Fermani, Lauren Velie, Francesca Sparla, Pierre Crozet, Julien Henri, Mirko Zaffagnini

**Affiliations:** ^1^ Department of Pharmacy and Biotechnologies, University of Bologna, Bologna, Italy; ^2^ Department of Chemistry “G. Ciamician”, University of Bologna, Bologna, Italy; ^3^ Interdepartmental Centre for Industrial Research Health Sciences & Technologies, University of Bologna, Bologna, Italy; ^4^ Department of Biochemistry and Molecular Biology, University of Massachusetts Amherst, Amherst, MA, United States; ^5^ Laboratory of Computational and Quantitative Biology, Sorbonne Université, CNRS, Institut de Biologie Paris-Seine, Paris, France

**Keywords:** carbon fixation, structure, catalysis, isomerase, epimerase, kinase

## Abstract

The Calvin-Benson-Bassham (CBB) cycle comprises the metabolic phase of photosynthesis and is responsible for carbon fixation and the production of sugar phosphates. The first step of the cycle involves the enzyme ribulose-1,5-bisphosphate carboxylase/oxygenase (Rubisco) which catalyzes the incorporation of inorganic carbon into 3-phosphoglyceric acid (3PGA). The following steps include ten enzymes that catalyze the regeneration of ribulose-1,5-bisphosphate (RuBP), the substrate of Rubisco. While it is well established that Rubisco activity acts as a limiting step of the cycle, recent modeling studies and experimental evidence have shown that the efficiency of the pathway is also impacted by the regeneration of the Rubisco substrate itself. In this work, we review the current understanding of the structural and catalytic features of the photosynthetic enzymes that catalyze the last three steps of the regeneration phase, namely ribose-5-phosphate isomerase (RPI), ribulose-5-phosphate epimerase (RPE), and phosphoribulokinase (PRK). In addition, the redox- and metabolic-based regulatory mechanisms targeting the three enzymes are also discussed. Overall, this review highlights the importance of understudied steps in the CBB cycle and provides direction for future research aimed at improving plant productivity.

## 1 Introduction

**Figure 1:**
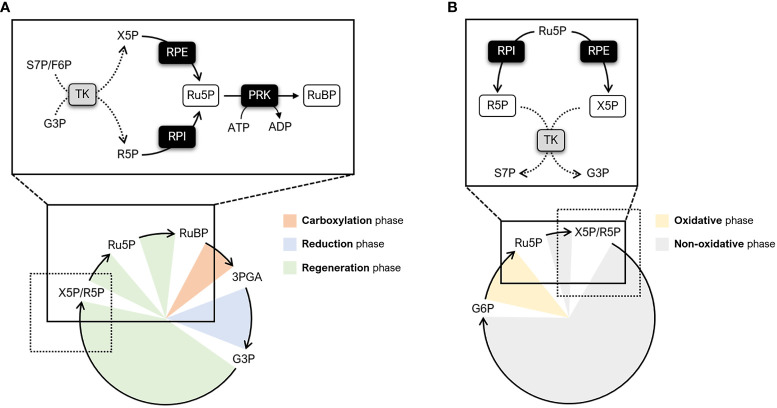
Schematic representation of metabolic pathways involving RPI, RPE, and PRK. **(A)** Simplified diagram of the CBB cycle highlighting the enzymatic steps catalyzed by RPI, RPE, and PRK (continuous lines). The different phases of the CBB cycle are indicated as follows: carboxylation phase (orange), reduction phase (light blue), and regeneration phase (light green). The reactions preceding RPI and RPE are indicated by dotted lines. **(B)** Simplified diagram of the PPP highlighting the enzymatic steps catalyzed by RPI and RPE (continuous lines). The different phases of the PPP are indicated as follows: oxidative phase (yellow) and non-oxidative phase (grey). The reactions following RPI and RPE are indicated by dotted lines. F6P, fructose-6-phosphate; G3P, glyceraldehyde-3-phosphate; G6P, glucose-6-phosphate; R5P, ribose-5-phosphate; Ru5P, ribulose-5-phosphate; RuBP, ribulose-1,5-bisphosphate; S7P, sedoheptulose-7-phosphate; TK; transketolase; X5P, xylulose-5-phosphate.

Primary biochemical production on Earth is mainly sustained by a complex metabolic pathway known as the Calvin-Benson-Bassham (CBB) cycle. The CBB cycle enables the fixation of atmospheric carbon dioxide (CO_2_) and thus plays a key role in plant metabolism by providing metabolic intermediates for starch and sucrose biosynthesis (Ruan, 2014; Pfister and Zeeman, 2016). Regardless of the photosynthetic organism (*e.g.*, cyanobacteria, algae, bryophytes, and land plants), this pathway is comprised on the same 11 enzymes and two regulatory proteins, namely the molecular chaperone Rubisco activase and the intrinsically disordered scaffold CP12 (Michelet et al., 2013; Bhat et al., 2017; Gerard et al., 2022). The first step of the CBB cycle involves the enzyme ribulose-1,5-bisphosphate carboxylase/oxygenase (Rubisco). Rubisco catalyzes the incorporation of CO_2_ into the 5-carbon sugar ribulose-1,5-bisphosphate (RuBP), yielding two molecules of 3-phosphoglyceric acid (3PGA). Based on the functional features of the enzyme, this Rubisco-dependent carboxylation reaction is considered a limiting step in the cycle as a whole (Andralojc et al., 2018). However, recent modelling studies and experimental evidence have highlighted that the efficiency of the CBB cycle is also co-limited by the regeneration of the Rubisco substrate (*i.e.*, RuBP) (Andralojc et al., 2018; Raines, 2022). Eight enzymes participate in the RuBP regeneration phase of the cycle by catalyzing intersecting reaction paths. In particular, five enzymes function cooperatively to transform the 3-carbon sugar and cycle product glyceraldehyde-3-phosphate (G3P) into two types of 5-carbon sugars, namely xylulose-5-phosphate (X5P) and ribose-5-phosphate (R5P). Following this step, the regeneration phase leads to the formation of ribulose-5-phospate (Ru5P) through separated reactions catalyzed by ribulose-5-phosphate 3-epimerase (RPE) and ribose-5-phosphate isomerase (RPI) (**Figure 1A**). Finally, the last step involves the enzyme phosphoribulokinase (PRK) which catalyzes the ATP-dependent conversion of Ru5P into RuBP (**Figure 1A**). While involved in the CBB cycle, RPI and RPE also participate in the non-oxidative branch of the pentose phosphate pathway (OPPP) catalyzing the reverse conversion of Ru5P into R5P and X5P, respectively (**Figure 1B**).

The aim of this review is to summarize the current understanding of the structural features of the photosynthetic enzymes RPI, RPE, and PRK, as well as their catalytic and regulatory properties with regard to their physiological role in the CBB cycle. We focused our work on these last three enzymatic steps of the CBB cycle because crystal structures of photosynthetic RPI, RPE, and PRK have recently been determined, thus allowing a better understanding of their structure/function relationship and their putative post-translational regulatory mechanisms (Gurrieri et al., 2019; Le Moigne et al., 2020; Meloni et al., 2022). Unless otherwise stated, we will describe the proteins from the model green microalga *Chlamydomonas reinhardtii*, from which the most extensive molecular data about the CBB cycle has been produced. Moreover, future perspectives relating to the biotechnological manipulation of these enzymes will be discussed. Globally, this work will help to generate a more complete overview of the carbon fixation pathway. Our emphasis on individual steps whose information in current literature is scarce or scattered aims to direct future research towards engineering strategies that have not yet been explored but which could positively impact the efficiency of photosynthetic carbon fixation.

## 2 Structural and biochemical features of plant ribose-5-phosphate isomerase

### 2.1 Plant RPI has a conserved fold shared with metabolic isomerases

**Figure 2:**
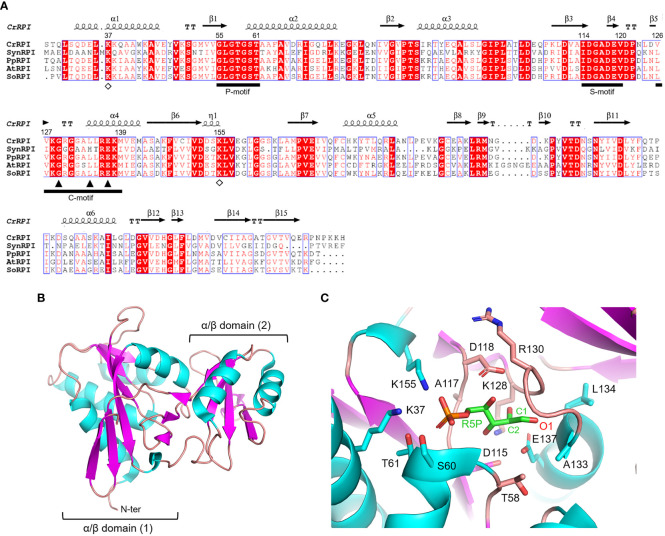
Sequence analysis and structural features of chloroplast ribose phosphate isomerase. **(A)** Multiple sequence alignment of RPI isoforms from model organisms was performed with ESPript (http://espript.ibcp.fr) using *Chlamydomonas reinhardtii* (Cr) RPI (PDB ID: 6ZXT; UNIPROT: A8IRQ1), *Synechocystis* PCC 6803 (Syn) RPI (UNIPROT: Q55766), *Physcomitrium patens* (Pp) RPI (UNIPROT: A9TF97), *Arabidopsis thaliana* (At) RPI (UNIPROT: Q9C998), and *Spinacia oleracea* (So) RPI (UNIPROT: Q8RU73). For the sake of clarity, the alignment was initiated by variably omitting the N-terminal residues and residue numbering is based on the sequence of CrRPI. Conserved residues are highlighted in white on red boxes, whereas residues with similar physicochemical properties are written in red on white boxes. Residues involved in substrate stabilization and catalysis are indicated by open diamonds and closed triangles, respectively. Ligand binding motifs are indicated by black bars as follows: phosphate binding motif (P-motif), sugar binding motif (S-motif), and catalytic motif (C-motif). The sequence identities were calculated with Clustal Omega (https://www.ebi.ac.uk/Tools/msa/clustalo/). **(B)** Main chain topology of CrRPI1 (PDB ID: 6ZXT) colored according to secondary structure types (α-helix: cyan, β-strand: magenta). **(C)** CrRPI1 catalytic site modelled with R5P from PDB ID 6MC0 (RPI from *Legionella pneumophila)*. Putative catalytic residues side chains are displayed as sticks.

Ribose-5-phosphate isomerase (RPI; EC 5.3.1.6) is a ubiquitous enzyme present in all living organisms with a relatively high degree of sequence conservation in photosynthetic model organisms (~40-68% of sequence identity) (**Figure 2A**). To date, structural data on RPI enzymes from non-plant organisms are rather extensive with more than fifty experimental 3D-structures available in the PDB repository. For instance, crystal structures have been determined for RPI from a variety of different organisms such as the bacterium *Escherichia coli* (PDB ID: 1LKZ) (Rangarajan et al., 2002), the unicellular eukaryote *Saccharomyces cerevisiae* (PDB ID: 1XTZ) (Graille et al., 2005), the archaeon *Pyrococcus horikoshii* (PDB ID: 1LK5) (Ishikawa et al., 2002), and numerous human parasites including the protozoan *Plasmodium falciparum* (PDB ID: 2F8M) (Holmes et al., 2006) and the euglenoid *Trypanosoma cruzi* (PDB ID: 3M1P) (Stern et al., 2011). In contrast, structural data of plant RPI isoforms is limited, and only the crystallographic structure of RPI from the model green alga *Chlamydomonas reinhardtii* (CrRPI, PDB ID: 6ZXT) has been experimentally determined (Le Moigne et al., 2020).

Photosynthetic RPIs belong to a common type A group also found in bacteria, archaea, and eukaryotes, while a structurally distinct type B is found only in parasitic protozoa and some bacteria. RPIs belonging to type A typically exist as homodimers, in which each monomer folds into two distinct α/β domains (**Figure 2B**). The catalytic domain is located at the N-terminus of the protein and belongs to the SCOPe structural family c.124.1.4, CATH classification 3.40.50.1360. The domain is composed of a six-strand β-sheet flanked by four α-helices and is connected to the carboxy-terminal lid domain through a short β-sheet (**Figure 2B**). The lid domain is composed of a four-strand antiparallel β-sheet and two α-helices and contributes to the formation of the homo-dimeric structure, which has been validated *in vitro* with recombinant CrRPI (Le Moigne et al., 2020).

Proper positioning of the RPI active site has been ascertained by alignment with the RPI structure from *Legionella pneumophila* (LpRPI, PDB ID: 6MC0) co-crystallized with both R5P and Ru5P. In detail, three conserved ligand-binding motifs were identified: the phosphate binding P-motif, the sugar binding S-motif, and the catalytic C-motif (**Figure 2A**). The structural alignment of CrRPI with the LpRPI:R5P complex allowed for the identification of 13 putative catalytic residues within the ligand-binding motifs that position side chains at a distance of 4 Å from the substrate (**Figure 2C**). In particular, Lys37 and Lys155 (numbering of mature chloroplastic CrRPI, **Figure 2A**) contribute positive charges for the binding of substrate phosphate group(s) and are perfectly conserved in other RPI isoforms from both plant and non-plant sources (**Figure 2A**). Relevant insights into structural features related to catalysis were obtained by analysis of the crystal structure of RPI from *Thermus thermophilus* HB8 (PDB ID: 1UJ5) in complex with R5P (Hamada et al., 2003). In this study, the authors revealed that substrate isomerization is stereospecific and proceeds through a *cis*-*ene*-diolate intermediate, in which the negatively charged O1 is stabilized by an oxyanion hole composed of the backbone amide nitrogens in the conserved Gly100-Leu105 motif (Gly129-Leu134 in CrRPI) (Hamada et al., 2003). Moreover, the conserved Glu108 (Glu137 in CrRPI, **Figure 2A**) is proposed to shuttle a proton from substrate C2 to C1, sequentially acting as both a general base and acid. Overall, these structural features are not exclusive to RPIs but are common to enzymes that catalyze isomerization reactions such as triosephosphate isomerase (TPI) and phosphoglucose isomerase (PGI) (Topper, 1957; Rose and O'Connell, 1960; Hamada et al., 2003).

### 2.2 Biochemical properties of RPI reveals that the direction of the reaction depends on the concentration of substrates

In photosynthetic organisms, RPI isoforms are found in both the cytoplasm and chloroplast (plastid) and are known to participate in the OPPP and the CBB cycle (**Figure 1**). In the CBB cycle, RPI plays an essential role by catalyzing the reversible conversion of R5P into Ru5P, an intermediate for the regeneration of RuBP, and is also involved in the formation of riboflavin and guanosine 5′-triphosphate (Volk and Bacher, 1991). When participating in the OPPP, RPI catalyzes the opposite reaction (*i.e.*, the conversion of Ru5P into R5P), which provides R5P later used for the synthesis of amino acids (*i.e.*, histidine and tryptophan) as well as purine and pyrimidine nucleotides (*e.g.*, nicotinamide dinucleotide, NAD) (Hove-Jensen, 1988). Despite its essential role in plant metabolism, there are currently few biochemical studies on plant RPI aimed at determining the catalytic mechanism, kinetic parameters, and possible regulatory mechanisms (*e.g.*, allosteric regulation and post-translational modifications (PTM)). Thus far, only chloroplast RPIs from pea and spinach have been characterized. Biochemical studies have revealed that pea RPI is capable to catalyze its reaction through a broad pH range with peak activity around 7.8, a value that corresponds to the stromal pH value under light conditions. In addition, the pea RPI isoform exhibits a similar affinity for both substrates, with a *K*_m_ of 0.9 and 0.6 mM for R5P and Ru5P, respectively (Skrukrud et al., 1991). Another study, however, reported a ~2.4-fold higher *K*_m_ for R5P (*K*_m,R5P_ = 2.2 mM; (Anderson, 1971)). The spinach isoform has proven to be similar to the pea homologue, showing a similar affinity for R5P, with a reported *K*_m_ of 0.63 or 0.46 mM (Rutner, 1970; Jung et al., 2000).

As mentioned previously, the interconversion reaction of Ru5P to R5P occurs through a catalytic mechanism shared with other isomerases (*i.e.*, TPI and PGI). The reaction consists in a single acid-base mechanism that involves a proton transfer between the carbon 1 and carbon 2 of the substrate *via* the formation of a *cis*-*ene*-diol(ate) intermediate (Topper, 1957; Rose and O'Connell, 1960). Configurational analyses and mutational studies have highlighted two highly conserved residues, located in the catalytic motif, as fulfilling a pivotal role in this process (Ishikawa et al., 2002; Hamada et al., 2003). Specifically, a conserved glutamate (Glu108 and Glu137 in *T. thermophilus* RPI and CrRPI, respectively, **Figure 2A**) is proposed to mediate the proton transfer between O1 and O2 through action as a catalytic base/acid. The residue Lys99 (Lys128 in CrRPI) seems to facilitate this process by orienting the glutamate side chain through salt bridges and thus contributing to its reactivity.

Based on its metabolic function, we can affirm that plant RPI plays a crucial role in the partitioning of pentose phosphates. It is therefore critical to ascertain the factors controlling the direction of this reaction. In this regard, equilibrium constants (*K*_eq_) of the enzyme have been determined and subsequently revealed that the reaction of RPI is near equilibrium (Skrukrud et al., 1991; Zhang et al., 2003). This suggests that, regardless of the metabolic process in which RPI is involved, the direction of the isomerization reaction is essentially driven by the concentration of the substrates. Therefore, whether RPI operates as an anabolic or catabolic enzyme depends on specific metabolic circumstances and/or the active regulation of other enzymes in the metabolic pathway.

## 3 Structural and biochemical properties of plant ribose-5-phosphate epimerase

### 3.1 Plant RPE is a metalloenzyme with a conserved TIM-barrel fold

**Figure 3:**
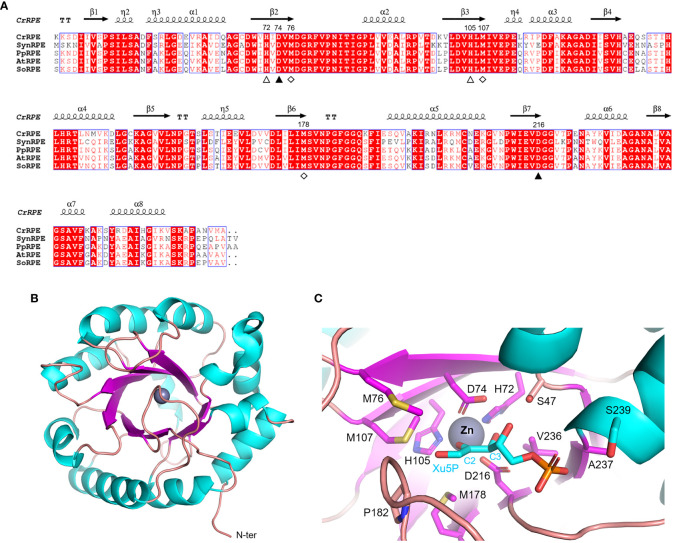
Sequence analysis and structural features of chloroplastic ribulose phosphate epimerase. **(A)** Multiple sequence alignment of RPE isoforms from model organisms was performed with ESPript (http://espript.ibcp.fr) using *Chlamydomonas reinhardtii* (Cr) RPE (PDB ID: 7B1W; UNIPROT: A8IKW6), *Synechocystis* PCC 6803 (Syn) RPE (UNIPROT: P74061), *Physcomitrium patens* (Pp) RPE (UNIPROT: A9RCK8), *Arabidopsis thaliana* (At) RPE (UNIPROT: Q9SAU2), and *Spinacia oleracea* (So) RPE (UNIPROT: Q43157). For the sake of clarity, the alignment was initiated by variably omitting the N-terminal residues and residue numbering is based on the sequence of CrRPE. Conserved residues are highlighted in white on red boxes, whereas residues with similar physicochemical properties are written in red on white boxes. Residues involved in catalytic zinc atom coordination are indicated by closed and open triangles, with the latter also involved in catalysis. Residues involved in the stabilization of the catalytic intermediate are indicated by open diamonds. The sequence identities were calculated with Clustal Omega (https://www.ebi.ac.uk/Tools/msa/clustalo/). **(B)** Main chain topology of CrRPE1 (PDB ID: 7B1W) colored according to secondary structure types (α-helix: cyan, β-strand: magenta). **(C)** CrRPE1 catalytic site modelled with X5P from PDB ID 3OVR (RPE from *Homo sapiens*). Putative catalytic residues side chains are displayed as sticks.

Ribulose-5-phosphate 3-epimerase (RPE, EC 5.1.3.1) is broadly distributed in all living organisms with a very high degree of sequence conservation in photosynthetic model organisms (64-90% identity) (**Figure 3A**). Due to its critical role in carbon-related pathways, RPE has been the subject of extensive structural analyses including the determination of crystal structures of the *Homo sapiens* enzyme (PDB ID: 3OVR) (Liang et al., 2011), RPE isoforms from the apicomplexan *Plasmodium falciparum* (PDB ID: 1TQX) (Caruthers et al., 2006), and from the pathogenic bacterium *Neisseria gonorrhoeae* (PDB ID: 5UMF). Crystal structures of RPE from photosynthetic organisms including *Synechocystis* (SynRPE, PDB ID: 1TQJ) (Wise et al., 2004), *Oryza sativa* (OsRPE, PDB IDs: 1H1Y and 1H1Z) (Jelakovic et al., 2003), *Solanum tuberosum* (StRPE, PDB ID: 1RPX) (Kopp et al., 1999), and *Chlamydomonas reinhardtii* (CrRPE, PDB ID: 7B1W) (Meloni et al., 2022) have also been determined. The only representatives of photosynthetic paralogues are StRPE and CrRPE. When considering all available structures from plant sources, we observed that chloroplast RPE assembles as homo-hexameric structures comprised of trimers of dimers. In contrast, cytoplasmic OsRPE has a dimeric fold as observed in human RPE, which was shown to behave allosterically through a possible monomer-dimer exchange (Karmali et al., 1983).

Regardless of the source organism however, RPE folds as a single (α/β)_8_-barrel domain of the triose phosphate isomerase family (TIM barrel, SCOPe c.1.2.2, CATH classification 3.20.20.70) (**Figure 3B**) and is generally considered a metalloenzyme. Two pairs of conserved His and Asp residues (His72, Asp74, His105, and Asp216 in CrRPE, **Figure 3A**) contribute to the stabilization of the metal ion within the active site (Akana et al., 2006) (**Figure 3C**). Purified RPE from *Escherichia coli* has been shown to be active *in vitro* when bound to various transition metals such as Mn^2+^, Co^2+^, Fe^2+^, or Zn^2+^, the latter giving a markedly lower catalytic constant (Sobota and Imlay, 2011). Human RPE co-crystallized with Fe^2+^, while RPE from *Streptococcus pyogenes*, *Oryza sativa*, and *Chlamydomonas reinhardtii* co-crystallized with Zn^2+^. The metal ion is proposed to be required for catalysis and is bound on top of the β-barrel, surrounded by side chains of eight residues which contribute to the substrate binding pocket (Liang et al., 2011) (**Figure 3C**). Structural evidence supports the proposed theory that the catalytic interconversion of X5P/Ru5P proceeds through an acid-base mechanism involving proton abstraction and donation and is proposed to occur through the formation of a *cis-ene-*diolate intermediate as aforementioned for RPI (Kopp et al., 1999). In the deprotonation-protonation mechanism, the two conserved Asp residues (Asp74 and Asp216 in CrRPE) play a fundamental role acting as a general base to deprotonate Xu5P/Ru5P at the carbon 3 and as well as functioning as a general acid to protonate the *cis-ene*-diolate intermediate (Jelakovic et al., 2003). The stabilization of the high energy intermediate involves the metal ion along with three conserved Met residues (Met76, Met107, and Met178 in CrRPE, **Figure 3A–C**). While the metal ion specifically interacts with carbon 2 and carbon 3 hydroxyl oxygens, the methionine residues bridge to the negative charged carbon 2 oxyanion (C2-O^–^). The methionine-dependent stabilization is guaranteed by the structural geometry and positioning of the three residues which globally act as proton-free sulfur cushion preventing isomerization, and thus favoring epimerization.

### 3.2 Biochemical analyses of plant RPE highlight large discrepancy in kinetic properties

As mentioned for RPI, RPE is also an amphibolic enzyme that participates in both the OPPP and the CBB cycle by catalyzing the reversible interconversion of X5P to Ru5P (*i.e.*, epimerization from Ru5P to X5P and from X5P to Ru5P in the OPPP and CBB cycle, respectively). Therefore, RPE plays a key role in partitioning pentose phosphates between these two essential metabolic pathways which coexist in the chloroplast of photosynthetic organisms (Schnarrenberger et al., 1995).

The evolutionary conservation of functionally essential residues (**Figure 3A**), most of which are found in the active site, indicates that RPE from different sources has specific structural elements that allow it to follow the same reaction mechanism (*e.g.*, conserved aspartate and methionine residues involved in the substrate to product transformation and the stabilization of the reaction intermediate, respectively (Jelakovic et al., 2003). To date, biochemical studies carried out on RPE from plant and non-plant sources specifically focused on the OPPP-related reaction (Ru5P conversion to X5P) highlighting a high degree of variability with regards to both substrate affinity and catalytic proficiency. Regarding plant isoforms, different turnover numbers (*k*_cat_) were estimated for the spinach enzyme and found to be 105-7100 sec^−1^ for the recombinant form and 0.138 sec^−1^ for the enzyme extracted from leaf chloroplasts, respectively (Chen et al., 1998; Teige et al., 1998; Meloni et al., 2022). Similarly, the affinity of spinach RPE for the substrate Ru5P is highly variable, with *K*_m_ values found to be 0.22, 0.25, and 1.56 mM (Chen et al., 1998; Teige et al., 1998; Meloni et al., 2022). Recent data obtained from recombinant CrRPE revealed a *k*_cat_ equal to 273 sec^−1^ and a *K*_m_ value for Ru5P of 1.52 mM (Meloni et al., 2022). The reason for these observed discrepancies in catalytic properties is uncertain and may depend on the intrinsic dissimilarity of the specific physiological context in which the plant enzymes operate, or on methodological differences during sample preparation and/or activity measurements through coupled enzymatic assays.

The conversion of X5P to Ru5P (CBB cycle-related activity) has only been investigated by a single study that analyzed the catalytic properties of recombinant CrRPE (Meloni et al., 2022). This data permitted a kinetic comparison with the enzymatic conversion of Ru5P to X5P (*i.e.*, OPPP-related reaction) while unravelling biochemical features of the enzyme that influence its metabolic role in the context of photosynthesis. Notably, the algal RPE has a turnover number of 80.7 sec^−1^, a value that is ~3-fold lower compared to the conversion of Ru5P to X5P, and a ~2-fold higher affinity for X5P with respect to Ru5P (*K*_m,X5P_ of 0.72 mM). The derived catalytic efficiencies (*k*_cat_/*K*_m_) are similar in comparison, being 1.13 x 10^5^ M^−1^ sec^−1^ and 1.79 x 10^5^ M^−1^ sec^−1^ (epimerization of X5P or Ru5P, respectively), which indicate that the two reactions are equally favored and that the direction of reaction depends on the concentration of the respective substrate.

## 4 Structural and biochemical features of plant phosphoribulokinase

### 4.1 Structural analysis of plant PRKs reveals a nucleoside hydrolase fold with conserved catalysis-related structural elements

**Figure 4:**
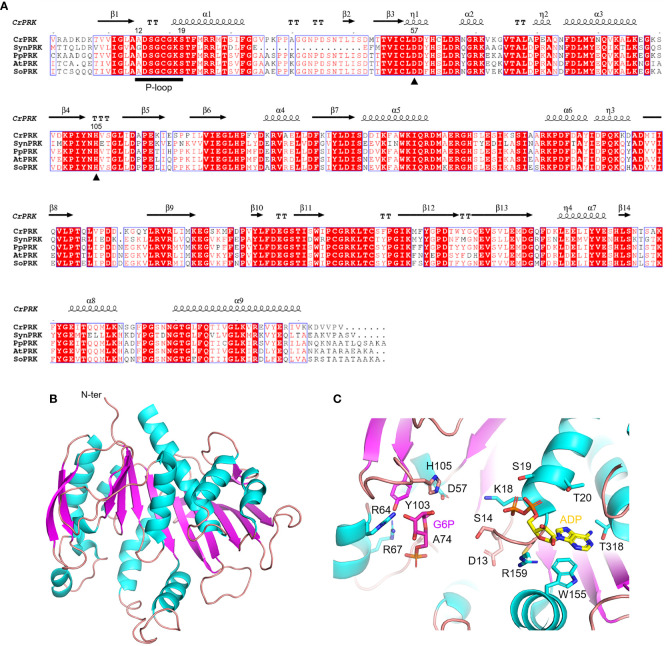
Sequence analysis and structural features of chloroplastic phosphoribulose kinase **(A)** Multiple sequence alignment of plant-type PRK isoforms from model organisms was performed with ESPript (http://espript.ibcp.fr) using *Chlamydomonas reinhardtii* (Cr) PRK (PDB ID: 6H7G; UNIPROT: P19824), *Synechocystis* PCC 6803 (Syn) PRK (UNIPROT: P37101), *Physcomitrium patens* (Pp) PRK (UNIPROT: A9TRN4), *Arabidopsis thaliana* (At) PRK (UNIPROT: P25697), and *Spinacia oleracea* (So) PRK (UNIPROT: P09559). For the sake of clarity, the alignment was initiated by variably omitting the N-terminal residues and residue numbering is based on the sequence of CrPRK. Conserved residues are highlighted in white on red boxes, whereas residues with similar physicochemical properties are written in red on white boxes. Residues acting as catalytic dyad are indicated by closed triangles. The ATP binding domain (P-loop) is indicated by black horizontal bar. The sequence identities were calculated with Clustal Omega (https://www.ebi.ac.uk/Tools/msa/clustalo/). **(B)** Main chain topology of CrPRK 3D-structure colored according to secondary structure types (α-helix: cyan, β-strand: magenta). **(C)** CrPRK catalytic site with ligands (ADP and glucose-6-phosphate) from PDB ID 6KEV (PRK from *Synechococcus elongatus* PCC 7942). Putative catalytic residues side chains are displayed as sticks.

The PRK enzyme is found exclusively in photosynthetic organisms (*i.e.*, cyanobacteria, algae, and land plants) and methanogenic archaea, and is involved in the CBB cycle and hexulose-phosphate reductive pathway, respectively (Kono et al., 2017). In these organisms, the primary sequence of PRK isoforms is variably conserved, ranging from 23% to 75%, with homology around 70% in the green lineage (**Figure 4A**). The first reported crystal structure of PRK originated from the purple photosynthetic bacterium *Rhodobacter sphaeroides* (PDB ID: 1A7J) (Harrison et al., 1998), while more recently the 3D-structures have been reported for the archeon *Methanococcus hungatei* (PDB ID: 5B3F), *Arabidopsis thaliana* (PDB IDs: 6H7H and 6KEX), *Chlamydomonas reinhardtii* (PDB ID: 6H7G), and for the cyanobacterium *Synechococcus elongatus* PCC 7942 (PDB IDs: 6HZK, 6H7L, and 6KEV) (Kono et al., 2017; Gurrieri et al., 2019; Wilson et al., 2019; Yu et al., 2020).

PRK belongs to the subgroup of nucleoside monophosphate (NMP) kinase superfamily characterized by an ATP binding domain (*i.e.*, P-loop) (**Figure 4A**), and can be distinguished into 3 types: archaeal-type (archaea), bacterial-type (α-cyanobacteria and proteobacteria), and plant-type (vast majority of cyanobacteria and plant eukaryotes) (Tabita, 1999; Stanley et al., 2013; Wilson et al., 2019). At the structural level, we recognized different oligomeric states based on PRK types. Notably, plant-type PRKs from *S. elongatus*, *A. thaliana* and *C. reinhardtii*, and the archeal-type from *M. hungatei* exhibit a dimeric folding, while the bacterial-type PRK from the α-proteobacterium *R. sphaeroides* is octameric (Harrison et al., 1998; Kono et al., 2017; Gurrieri et al., 2019; McFarlane et al., 2019; Yu et al., 2020). Within the oligomer, the interface area between monomers has decreased during the proteins’ evolution (from bacterial- to plant-type passing through archaeal-type) with possible implications on structure flexibility (Gurrieri et al., 2019).

The PRK fold belongs to SCOPe family c.37.1.6, and is related to CATH classification 3.40.50.300 of the Rossman fold. Focusing on the plant-type, PRK folds as a remarkable extended 10-strand mixed β-sheet sided by a 2-strand antiparallel β-sheet. Nine α-helices dock onto the sides of the main β-sheet (**Figure 4B**). The last β-strands of each subunit converge to establish the monomer interface through multiple contacts (Gurrieri et al., 2019). The precise location of the active site in the PRK enzyme was obtained by analyzing the 3D-structure of *A. thaliana* PRK co-crystallized with the reaction product ADP and an analogue of the Ru5P substrate, namely glucose-6-phosphate (G6P) (Yu et al., 2020). This analysis allowed for the identification of protein residues contributing to the interaction with the cofactor and substrate within the active site (**Figure 4C**). In addition, the conserved catalytic dyad involving an Asp and a His residue was also identified (Asp58 and His106 in AtPRK, corresponding respectively to Asp57 and His105 in CrPRK) **Figures 4A–C**), whose interaction enables deprotonation of the acidic residue. This proton exchange allows Asp57 (CrPRK numbering) to serve as a catalytic base, thus activating the transfer of the phosphate group from ATP to Ru5P (see following for further details). Another conserved structural element is the P-loop (**Figures 4A–C**). As aforementioned, this region of the protein is fundamental to the binding of ATP placing the γ-phosphate of ATP close to carbon 1 of the sugar substrate, *i.e.*, Ru5P (Yu et al., 2020). However, as observed for other protein kinases, proper ATP binding is only guaranteed in the presence of the nucleoside triphosphate complexed to magnesium ions, which, however, is not visible in any of the reported structures. Other bivalent cations (*e.g.*, Mn^2+^ and Ca^2+^) can substitute Mg^2+^, although yielding suboptimal catalysis (Hurwitz et al., 1956; Marsden and Codd, 1984), suggesting possible alteration of activity as a consequence of changes in ion levels *in vivo*.

### 4.2 Biochemical features of plant PRK disclose similar affinity towards substrate and ATP

In the CBB cycle, PRK catalyzes the ATP-dependent phosphorylation of carbon 1 of Ru5P yielding RuBP, ADP, and inorganic phosphate. During the catalytic mechanism, the active site Asp57, upon interaction with a histidine that causes its deprotonation, functions as a catalytic base and activates carbon 1 of Ru5P. This activated carbon in turn initiates a nucleophilic attack on the γ-phosphoryl group of ATP, thus leading to the phosphorylation of Ru5P to RuBP (Harrison et al., 1998; Yu et al., 2020). To ensure efficient catalysis, substrates are bound in sequential order. First, ATP enters the active site and induces a conformational rearrangement that allows for Ru5P binding (Lebreton and Gontero, 1999; Yu et al., 2020). This conformational change also prevents unwanted hydrolysis of ATP that might occur in an environment otherwise accessible to the stromal milieu rich in pentose phosphate sugars other than Ru5P (Schlauderer et al., 1996; Sigrell et al., 1998; Li et al., 2004).

The affinities for the substrate and cofactor cover similar ranges. The *K*_m_ for Ru5P of plant PRK (*i.e.*, cyanobacterial, algal and land plant isoforms) has been reported to vary from 50 to 270 μM and the affinity for Mg^2+^-ATP similarly ranges from 34 to 280 μM (Hurwitz et al., 1956; Anderson, 1973; Surek et al., 1985; Roesler and Ogren, 1990; Wadano et al., 1995; Hariharan et al., 1998; Kobayashi et al., 2003; Michels et al., 2005; Thieulin-Pardo et al., 2015). Likewise, specific activities reported for the different plant-type PRKs are comparable ranging from 218 to 588 µmol min^−1^ mg^−1^, values that are significantly higher than those found for archeal and many bacterial PRK types (1.68-111 µmol min^−1^ mg^−1^) (Tabita, 1988; Kono et al., 2017). These differences are likely related to the structural diversity of PRKs (see previous) and could underlie the variability of metabolic fluxes through carbon fixation pathways in the different organisms in which PRK is found.

## 5 Regulatory mechanisms of RPI, RPE, and PRK

### 5.1 Thiol-switching regulatory control of RPI, RPE, and PRK catalysis

It is well known that protein thiols play a central role in signaling pathways as they are known to couple a change in intracellular redox state to biochemical responses. In response to redox signals, protein cysteine thiols can undergo different types of redox modifications such as disulfide bond formation (−SS−), oxidation (*i.e.*, sulfenylation −SOH, sulfinylation −SO_2_H, sulfonylation −SO_3_H), S-glutathionylation (−SSG), and S-nitrosylation (−SNO). Based on numerous proteomic studies, hundreds of chloroplast proteins have been identified putative targets of cysteine-dependent redox modifications (Zaffagnini et al., 2019) and references therein). Intriguingly, it has been observed that all CBB cycle enzymes are likely regulated by thioredoxin-mediated disulfide bond reduction, S-glutathionylation, and S-nitrosylation (Zaffagnini et al., 2019) and references therein). Apart from the well-established TRX-dependent regulation of four CBB cycle enzymes through dithiol/disulfide interchanges (Michelet et al., 2013), experimental demonstration for activity modulation *via* redox modifications of the remaining CBB cycle enzymes is far from complete.

In the following section, we will focus our attention on the current knowledge of the redox-based regulatory mechanisms of the three CBB enzymes discussed throughout this review: RPI, RPE, and PRK.

**Figure 5:**
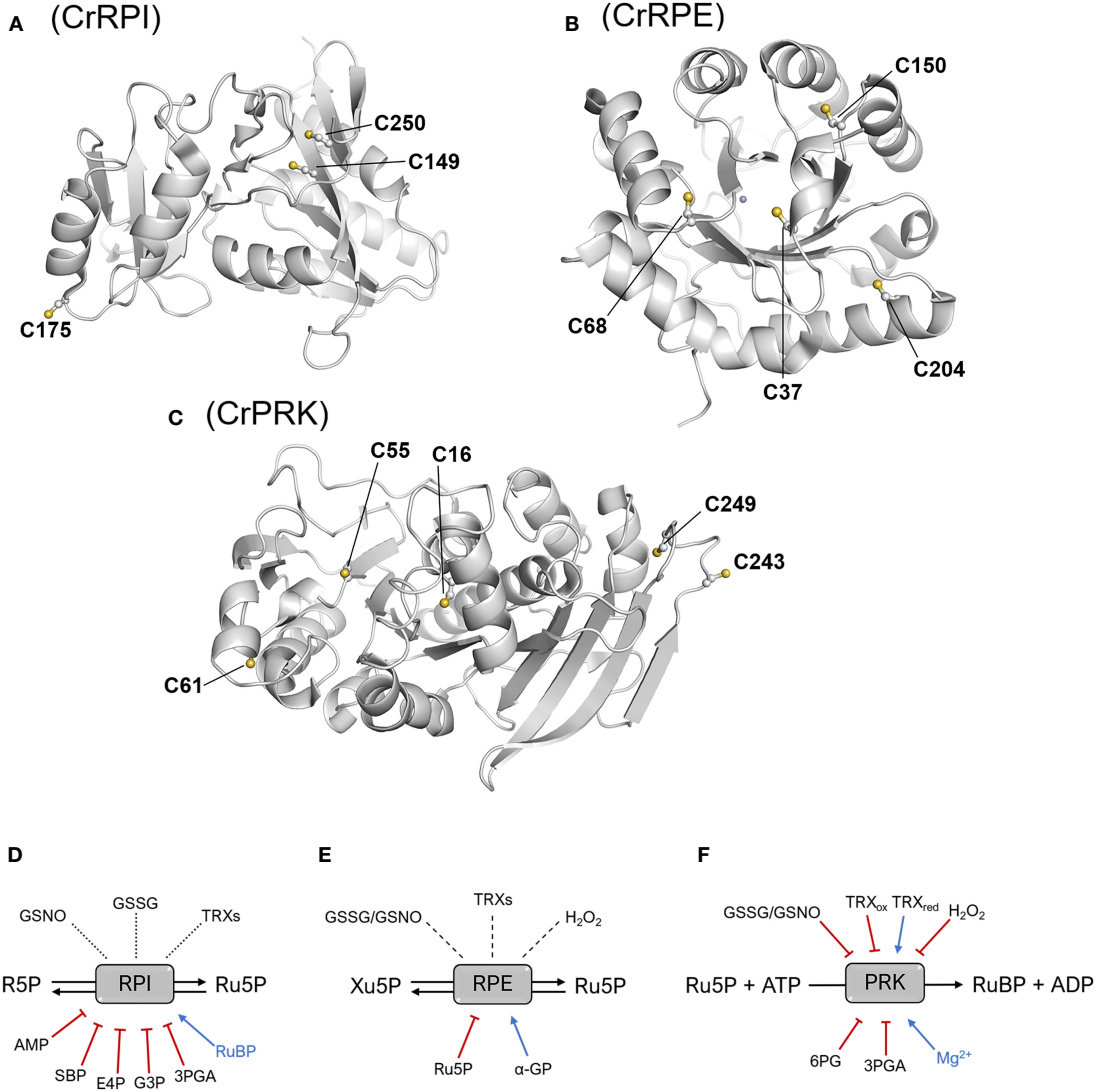
Structural position of cysteine residues and thiol- and metabolic-based regulatory mechanisms. Structure of the monomers of CrRPI **(A)**, CrRPE **(B)**, and CrPRK **(C)** highlighting cysteine residues involved in established or putative regulatory mechanisms. Established or putative regulatory mechanisms of plant RPI **(D)**, RPE **(E)**, and PRK **(F)**. Blue arrows and red barred lines indicate molecules/metabolites that cause inhibition/destabilization or enhancement of enzyme activity, respectively. Dotted lines designate putative thiol switching regulation as suggested by redox-based proteomic studies, while dashed lines indicate that the redox sensitivity of enzymes has been evaluated, but no effect on the catalysis was determined. 3PGA, 3-phosphoglycerate; 6PG, 6-phosphogluconate; α-GP: α-glycerophosphate; AMP, adenosine monophosphate; E4P, erythrose-4-phosphate; G3P, glyceraldehyde-3-phosphate; GSNO, S-nitrosoglutathione; GSSG, oxidized glutathione; Ru5P, ribulose-5-phosphate; SBP, sedoheptulose-1,7-bisphosphate; TRXs, thioredoxins; TRX_ox/red_, oxidized/reduced thioredoxin.

Although RPI has been identified as a target of oxidative modifications, there are currently no biochemical studies that investigate possible redox regulation. However, the crystallographic structure of CrRPI has allowed us to derive molecular hints about cysteine thiols that may serve to guide future biochemical studies (**Figure 5A**). In particular, Cys149 and Cys250 are positioned at a distance suitable for the formation of an intramolecular disulfide bond (**Figure 5A**), although their buried position would require local conformational changes to allow the interaction with redox regulators such as thioredoxins. At the same time, the sulphur atom of Cys175 is the most exposed to the solvent (**Figure 5A**), which could facilitate the reaction with oxidative molecules (*e.g.*, hydrogen peroxide and nitrosoglutathione), thus making it the most likely target of redox modifications (Le Moigne et al., 2020). However, biochemical evidence of this regulation remains insufficient.

Analogously to CrRPI, the 3D-structure of RPE from *C. reinhardtii* also offers suggestions for cysteine thiols sensitive to redox regulation. CrRPE contains four cysteines, but structural evidence has not revealed thiol groups prone to form disulfide bonds (**Figure 5B**). Consistently, no modulation in activity was observed after treatments with thioredoxin in combination with oxidized or reduced dithiothreitol (Meloni et al., 2022) (**Figure 5E**). Among CrRPE cysteines, only Cys37 was observed to be accessible to the solvent (**Figure 5B**), and therefore exists as a possible candidate for a regulatory role. However, oxidative agents (*i.e.*, nitrosoglutathione, oxidized glutathione, hydrogen peroxide), have been demonstrated to induce a negligible effect in terms of modulation of enzymatic activity (Meloni et al., 2022) (**Figure 5E**). To date, SoRPE proves to be the only isoform responsive to redox treatment, but the mechanism underlying the inhibitory effect induced by the reducing agent mercaptoethanol has not been elucidated (Chen et al., 1998).

Plant-type PRK is one of the four enzymes of the CBB cycle to be regulated by thioredoxins (Michelet et al., 2013; Gurrieri et al., 2021). The redox regulation of PRK mainly occurs during dark/light cycles through dithiol/disulfide exchange reactions involving two conserved cysteine pairs. Regulatory cysteine residues are located in the N-terminal and C-terminal portions of the protein (Cys16-Cys55 and Cys243-Cys249 in CrPRK) (**Figure 5C**). Under light conditions, reduced TRXs catalyze the Cys16-Cys55 disulfide reduction, relieving the structural constrains affecting proper ATP binding and thus triggering PRK catalytic activation (Marri et al., 2009; Gurrieri et al., 2019; Wilson et al., 2019) (**Figure 5F**). TRXs are also responsible for PRK inactivation at night (**Figure 5F**). Since RuBP is utilized only by the CBB cycle and not required by other metabolic pathways, this inactivation ensures the prevention of metabolite accumulation. Disulfide bond regulation of PRK works side by side with the formation of a supramolecular complex involving the regulatory protein CP12 and glyceraldehyde-3-phosphate dehydrogenase, another CBB cycle enzyme (Pohlmeyer et al., 1996; Wedel and Soll, 1998). Within the complex, PRK is almost completely inactivated (Graciet et al., 2004; Marri et al., 2008), and since the C-terminal Cys243-Cys249 disulfide of PRK is observed in all GAPDH-CP12-PRK complex structures (McFarlane et al., 2019; Yu et al., 2020), it is postulated to be important for complex assembly. Besides thioredoxin-dependent regulation, PRK has also been identified as a putative target of other redox modifications. A biochemical study highlighted that enzyme activity can be modulated *in vitro* by several redox agents like glutathione, nitrosoglutathione, and hydrogen peroxide (Marri et al., 2014), but the effective redox regulation mediated by these compounds remains to be confirmed *in vivo*.

In conclusion, we can affirm that while RPE proved to be irresponsive towards redox-based regulatory mechanisms, the redox regulation of RPI remains uncertain. In contrast, PRK activity is shown to be modulated by different types of redox modifications, confirming that it is an effective redox target in agreement with redox-based proteomics. It should be considered that redox post-translational modifications may, in actuality, not be implicated in the regulation of enzyme activity, but may instead impact protein function by promoting interactions with other proteins or by inducing “moonlighting” functions that are presently unknown (Zaffagnini et al., 2014). Further studies are essential to gain insight into the role of redox signals in modulating protein function and to decipher the physiological impact of these regulatory mechanisms on carbon fixation and related metabolic fluxes.

### 5.2 Metabolite- and cofactor-dependent regulation of RPI, RPE, and PRK (Regulation of RPI, RPE and PRK activity through metabolic-dependent mechanisms)

When examining the physiological context, it is worth considering the effect that metabolites may have in regulating the concerted activities of enzymes. To this end, we retrieved biochemical studies addressing the impact of certain molecules (*e.g.*, sugars, organic acids, and nucleotides) on the activity of CBB cycle enzymes. We found that there is an abundant amount of data regarding RPI, but little regarding RPE and PRK. In particular, pea RPI has been shown to be inactivated in a competitive manner by adenosine monophosphate (AMP), and various metabolites such as erythrose-4-phosphate (E4P), sedoheptulose-1,7-bisphosphate (SBP), glyceraldehyde-3-phosphate (G3P), and 3-phosphoglycerate (3PGA) (**Figure 5D**). The inhibitory effect of these molecules has been observed at concentrations close to those found in photosynthesizing chloroplasts, with constant inhibitions (*K*_I_) ranging from 0.1 to 1 mM (Anderson, 1971; Skrukrud et al., 1991). In agreement with these data, a similar inhibition response was observed for spinach RPI towards E4P and 3PGA (Woodruff and Wolfenden, 1979). Conversely, RuBP has been observed to have a positive effect on enzyme activity (Anderson, 1971; Skrukrud et al., 1991) (**Figure 5D**). In the case of RPE, only α-glycerophosphate and Ru5P have been tested and found to have stabilizing and destabilizing effects on enzyme activity, respectively (Chen et al., 1998) (**Figure 5E**). With regard to PRK, few studies have focused on evaluating possible metabolite-induced inhibition, but available data reveals different responses between PRK of cyanobacteria and that of land plants. Data on PRK from algae are not available. In cyanobacteria, AMP and ADP inhibit PRK at concentration between 0.32 and 1 mM (Marsden and Codd, 1984; Wadano et al., 1995), while the same response was not observed in PRK from land plants, with pea PRK activity appearing insensitive to both AMP and ADP (Anderson, 1973). Moreover, PRK from *Synechocystis* PCC 6803 exhibits strong sensitivity to isocitrate (Nishiguchi et al., 2020), while the impact of this compound on plant-type PRK is not known. The only metabolite that was found to affect PRK activity from both land plants and cyanobacteria is 6-phosphogluconate (Anderson, 1973; Wadano et al., 1995). This molecule is an intermediate of the OPPP and its impact on PRK catalysis is likely required to coordinate metabolic fluxes between the OPPP and the CBB cycle. Finally, it has been observed that 3-phosphoglycerate induces inhibition of PRK from wheat and pea (Anderson, 1973; Surek et al., 1985), but its role remains in question as protein inactivation was observed at concentrations above the physiological range (6 mM).

In addition to post-translational redox modifications and metabolic-dependent regulation, protein activity may also be modulated by other mechanisms such as serine/tyrosine phosphorylation. Notably, it is estimated that 30% of proteins in eukaryotic cells are subjected to this kind of PTM (Wang et al., 2014). In the model green alga *Chlamydomonas reinhardtii*, phosphoproteomic studies identified more than one-thousand proteins including RPI, RPE and PRK as putative targets. Regarding CrRPI and CrRPE, structural analysis suggested that the phosphorylation could prevent substrate entry into the active site, representing a possible mode of inhibitory regulation of these enzymes (Le Moigne et al., 2020; Meloni et al., 2022). To date, no structural details on putative phosphorylation sites are available for PRK. Despite being targeted by phosphorylation, there remains a lack of knowledge about the molecular mechanisms and physiological conditions that control the phosphorylation state of the three enzymes.

## 6 Conclusion and perspectives

### 6.1 Concluding remarks

Based on available data in previously published research and in databanks, this work summarizes the current knowledge on the structural and functional features of three CBB cycle enzymes participating in the last steps of the CBB cycle allowing the formation of Rubisco substrate, *i.e.*, RuBP. Detailed data on the catalytic and structural properties of plant RPI, RPE, and PRK are available for only a very few number of species. This scattered information does not allow for a complete understanding of the three CBB cycle enzymes relative to their specific physiological context.

### 6.2 Perspectives for future research

#### 6.2.1 Integration of the CBB cycle with metabolism

The interplay between the catabolic OPPP and the CBB cycle must be tightly controlled in order to limit futile cycling of R5P and X5P with Ru5P at the steps catalyzed by RPI and RPE. While *Chlamydomonas reinhardtii* possesses at least two genes coding for RPI and RPE, it would be of interest to attribute a metabolic function either to each enzyme isoform, or to post-translational modification states of the enzymes. Paralog matching to metabolic function has been explored by us and collaborators on the CBB cycle and glycolytic aldolases (Le Moigne et al., 2022). Alternatively, the direction of metabolites towards the CBB cycle or the OPPP may simply be controlled by their concentrations in the chloroplast.

#### 6.2.2 Increase photosynthetic carbon fixation by rational enzyme engineering

Despite the functional properties revealed thus far, the critical question we pose is whether there is a need and a possibility to rationally improve these functions in order to make individual enzymes more efficient and thus enhance the metabolic flux of the CBB cycle. In particular, catalytic proficiency and affinity toward substrate and/or cofactor appear suboptimal when compared with catabolic orthologs or considering physiological metabolites concentrations in the cell. To this end, site-directed mutagenesis of critical residues would, for example, allow for positive modulation of substrate binding and thus increase catalytic efficiency by decreasing the Michaelis-Menten constant for these interactions. Further studies of these enzymes and their catalytic properties would allow for the future potential to improve upon native characteristics and engineer a more efficient, more productive organism.

#### 6.2.3 Expand modelization to other plants

Experimental structures, functions, and regulation are only described for the green microalga *Chlamydomonas reinhardtii* RPI, RPE and PRK at the date of this review. In the future, it will be fundamental to extend our knowledge of the natural diversity of these enzymes and to better understand the relationship between the structure/function of the CBB cycle enzymes from diverse plant species including alternative model plants, crops, and metagenomic sequences (Villar et al., 2018). The catalytic and structural diversity of these three CBB cycle proteins originating from different organism must be analyzed alongside the primary sequences, in order to identify potential catalytic switches and the specific functional roles of the conserved and non-conserved amino acids. In this regard, experimental evidence and modelling studies have provided novel predictions on how to further enhance RuBP regeneration (Andralojc et al., 2018; Simkin et al., 2019; Raines, 2022).

#### 6.2.4 Systems and synthetic biology of the CBB cycle

Testing these outputs will require a multidisciplinary approach consisting of biochemical and structural studies coupled with quantitative analysis of the metabolic profile within relevant subcellular compartments. In addition, the application of new approaches to identify genetic factors and biochemical mechanisms involved in regulating the expression and activity of CBB cycle enzymes will benefit from the application of gene-editing technologies in order to modify each reaction step of the pathway. Remarkably, it may be possible to use synthetic biology to introduce improved enzymes that operate within the existing cycle or to replace whole branches of the pathway. The emergence of these new technologies offers researchers an attractive toolbox with which to engineer the full potential that improvements in RuBP regeneration can contribute to increasing photosynthetic performance and crop yield. Whether the enzymes examined in this work can be improved and whether such functional improvements can have an impact on photosynthesis and primary production is still an open but certainly interesting question to be tested.

## Author contributions

All authors were involved in the writing and revision of the manuscript, and approved the final manuscript.
